# Diabetic foot care and cardiovascular surgery in Peru: a looming crisis of centralism and unequal resource allocation

**DOI:** 10.47487/apcyccv.v6i4.576

**Published:** 2025-12-29

**Authors:** W. Samir Cubas

**Affiliations:** 1 Division of Cardiovascular Surgery, Department of Surgery, London Health Sciences Centre, Western University, London, Ontario, Canada. Western University Division of Cardiovascular Surgery, Department of Surgery London Health Sciences Centre Western University London, Ontario USA; 2 Harvard Medical School, Harvard University, Boston, United States. Harvard University Harvard Medical School Harvard University Boston USA

*Dear Editor,*

Diabetes mellitus and its vascular complications are growing public health challenges in Latin America, with Peru facing particularly severe structural inequities in access to specialized surgical care. Recent epidemiological evidence, including a systematic review and meta-analysis, estimated a national prevalence of diabetes of 7.47% in Peru. ^[^^1^^]^ Applying these estimates to the 2025 national population of 34,576,665 individuals yields approximately 2,582,877 people with diabetes and 3,685,872 with prediabetes. ^[^^2^^]^ International evidence consistently indicates that individuals with diabetes face a lifetime risk of 19% to 34% of developing diabetic foot complications, including ulcers and peripheral vascular disease requiring revascularization or amputation. ^[^^3^^]^ Extrapolating these figures to Peru, the expected lifetime diabetic foot burden ranges from 490,747 to 878,178 cases nationally.

Cardiovascular surgeons are key players in diabetic foot prevention; however, recent data obtained from the Medical College of Peru’s public registry (https://aplicaciones.cmp.org.pe/conoce_a_tu_medico/) show that the country has only 553 registered cardiovascular surgeons, equating to roughly one surgeon per 62,526 inhabitants. ^[^^4^^]^ If the national diabetic foot burden were evenly distributed, each surgeon would face an expected lifetime caseload of approximately 887 to 1,588 diabetic foot cases, in addition to their other cardiovascular responsibilities.

**Figure 1:**
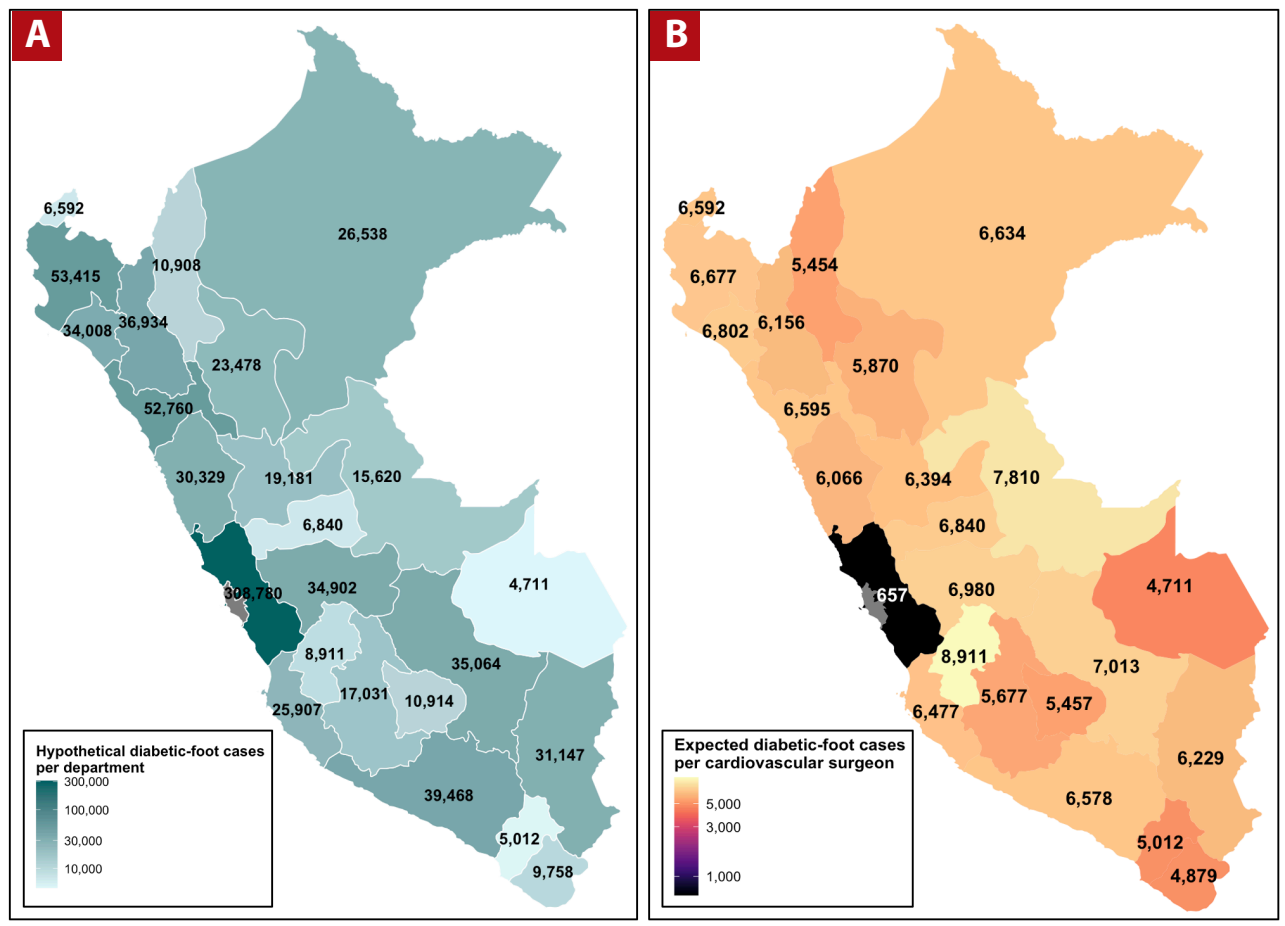
A. Diabetic Foot Distribution by Department: Estimated lifetime diabetic foot cases across Peruvian departments. B. Diabetic Foot Cases per Cardiovascular Surgeon: Expected lifetime diabetic foot cases per cardiovascular surgeon by department in Peru (upper-bound, 34% lifetime risk).

The problem is not merely the low absolute number of cardiovascular surgeons-it is the extreme geographic centralism of their distribution. Approximately 85% of these surgeons are based in Lima, the capital, which has a population of about 10.4 million inhabitants, representing only 30% of the national population. This leaves only 15% of cardiovascular surgeons to cover the remaining 70% of the population, dispersed across 24 regions. The implications of this maldistribution are striking. Lima’s 470 cardiovascular surgeons serve a population of 10.4 million, corresponding to roughly 22,128 inhabitants per surgeon. In contrast, the 83 surgeons working outside Lima must collectively serve 24,176,665 people, resulting in a staggering 291,285 inhabitants per surgeon-a 13-fold disparity. When these population ratios are applied to the diabetic foot burden, the inequities become even more evident. In Lima, the expected lifetime diabetic foot caseload per surgeon ranges from 314 to 657 cases. In the rest of the country, each surgeon faces between 5,012 and 8,911 cases over their professional lifetime, assuming uniform prevalence across regions (Figure 1). This estimate does not account for the additional challenges posed by the lower availability of diagnostic equipment, fewer multidisciplinary diabetic foot teams, limited endovascular capacity, and transportation barriers for rural patients. ^[^^5^^]^

Peru's extreme concentration of specialists in Lima stems from longstanding health policy centralism. Historic investment favored the capital, creating a virtuous cycle of better infrastructure, attracting more specialists justifying further investment. ^[^^6^^]^ Conversely, regions face a vicious cycle: limited resources and professional isolation discourage specialists, causing chronic under-resourcing. With no incentives for redistribution and cardiovascular surgery training centralized in Lima, graduates stay for better opportunities. ^[^^7^^]^ This forces many patients to travel for care, often resulting in delayed diabetic foot treatment and avoidable amputations.

In Peru, effective diabetic foot care requires early vascular assessment and multidisciplinary teams. However, regional hospitals often lack essential tools and vascular surgeons. This mismatch forces conservative treatment, delayed referrals, or preventable amputations, creating severe inequities where patients outside Lima face higher amputation rates and mortality. ^[^^8^^]^

Despite progress, Peru's healthcare faces challenges in diabetic foot care and cardiovascular surgery due to centralization, fragmented governance, and a lack of workforce planning and incentives for specialists outside Lima. However, optimism exists due to improved epidemiological data, a young generation of surgeons gaining international training, and technological advances like telemedicine. Targeted policy interventions and hub-and-spoke models can help correct regional imbalances. ^[^^3^^,^^5^^]^

In conclusion, Peru faces a preventable diabetic foot crisis due to rising diabetes, limited surgeons, and extreme centralization. Recommended actions include implementing a national diabetic foot strategy, incentives to redistribute surgeons, decentralizing training, investing in regional infrastructure, integrating telemedicine, and setting workforce targets. Strategic reforms can correct imbalances, ensuring geography no longer determines a patient’s fate and moving toward equitable, limb-saving care
